# Adropin: A New Regulator of Testicular Function—What Do We Know So Far?

**DOI:** 10.3390/ijms27104236

**Published:** 2026-05-10

**Authors:** Asmaa A. Muhammed, Rasha Babiker

**Affiliations:** 1 Department of Physiology, RAK College of Medical Sciences, RAK Medical & Health Sciences University, Ras-AlKhaimah 11127, United Arab Emirates; rashababiker@rakmhsu.ac.ae; 2 Department of Medical Physiology, Faculty of Medicine, Aswan University, Aswan 81528, Egypt

**Keywords:** adropin, spermatogenesis, testis, testosterone

## Abstract

Adropin, a peptide hormone first identified by microarray analysis of gene expression in mice’s liver, is expressed in multiple organs, including the brain, liver, and heart. It is a key regulator of carbohydrates and lipid metabolism. Moreover, it has anti-inflammatory and antioxidative effects. It enhances blood vessel dilation and is essential for the normal development and function of the cerebellum. It acts through binding to multiple receptors, primarily the orphan G protein-coupled receptor, which is expressed in various tissues, including the central nervous system, liver, heart, kidneys, and testis, suggesting a direct role of adropin in modulating the function of multiple organs. This review discusses recently identified testicular functions regulated by adropin. Some studies have demonstrated that adropin can stimulate testosterone synthesis in testicular Leydig cells by enhancing the expression of steroidogenic enzymes. Moreover, it increases germ cell proliferation and sperm formation by inhibiting apoptosis and oxidative stress. Current evidence remains limited, and further studies are required to clarify the underlying mechanisms of adropin in reproductive physiology. Moreover, its potential role in conditions associated with altered testosterone levels or impaired spermatogenesis remains speculative and requires validation in well-designed clinical studies.

## 1. Introduction

Kumar and his colleagues were the first to discover adropin during an analysis of liver gene expression in obese mice. The name “adropin” is derived from two Latin words: aduro (to set fire) and pinquis (fat) [1].

Adropin is formed of a 76-amino acid sequence, which is identical in humans, mice, and rats. The energy homeostasis-associated (Enho) gene encodes adropin. Adropin is expressed in the liver and the brain [1,2]. In addition, other organs such as the central nervous system, heart, kidney, liver, human umbilical vein, and pancreas synthesize adropin [3].

Kumar et al. [1] discovered that adropin gene expression in the liver is affected by diet. It is stimulated by feeding and decreased by fasting. In addition, high levels of adropin were found in high-fat-diet- and low-carbohydrate-diet-fed mice [4]. In humans, adropin correlated positively with fat consumption and negatively with carbohydrate consumption [5].

Several receptors have been identified as targets of adropin, such as the orphan G protein-coupled receptor (GPR19) that has been expressed in various tissues such as the hypothalamus, cerebellum, testis, heart, liver, and kidney [6]. The vascular endothelial growth factor receptor 2 (VEGFR2) also was identified as a potential adropin receptor in the endothelium through which adropin increases the endothelial nitric oxide synthase expression [7]. Additionally, adropin can enhance cerebellar development through acting on the brain NB-3/Contactin 6 receptor [2].

## 2. Metabolic, Anti-Inflammatory, and Antioxidant Effects of Adropin

Adropin peptide has an essential role in regulating lipid and glucose balance as well as improving insulin sensitivity [8,9,10]. It is involved in lipid metabolism, represented by diminishing total cholesterol (TC), triglycerides (TG), and low-density cholesterol level (LDL-c) [11].

In adropin knockout mice, Ganesh-Kumar et al. [4] observed dyslipidemia, dysregulation of endogenous carbohydrate synthesis, and insulin resistance despite maintaining normal food and energy consumption. Lower adropin levels are considered a risk factor for insulin resistance and dyslipidemia [12].

Adropin administration to diet-induced obese (DIO) mice increased glucose tolerance, improved insulin resistance, and promoted glucose oxidation [13]. Gao et al. [14] demonstrated that adropin, when given to DIO mice, reduced blood glucose through decreasing glucose production from the liver and enhancing insulin action.

Adropin influenced fuel selection preference in skeletal muscles. It promoted carbohydrate oxidation rather than fatty acids by activating pyruvate dehydrogenase in muscle [15]. It increased glucose transporter type 4 (GLUT4) cell-surface expression in muscles [13]. In the heart as well, adropin increased cardiac glucose oxidation in mice kept on long-term high-fat diets [16]. It enhanced insulin inhibition of fatty acid oxidation in the hearts of fasting mice, increasing cardiac glucose utilization and cardiac work and improving cardiac efficiency [17].

Lifelong caloric restriction in mice induces metabolic adaptation in the form of reduction in lipogenesis, increased lipolysis, and ketogenesis in the liver, which could be explained by the increase in adropin gene expression in the liver of these mice [18]. Adropin, when given to mice, markedly reduced plasma TG levels, hepatic TG synthesis, and expression of enzymes involved in de novo fatty acid synthesis and fatty acid oxidation [17].

The anti-inflammatory and antioxidative effects of adropin are well established [9,19,20]. Adropin administration to diabetic rats decreased tumor necrosis factor-α (TNF-α) mRNA expression in their pancreas [11]. Its administration suppressed TNF-α-induced monocyte adhesion to endothelial cells of the human umbilical vein and shifted macrophages to the anti-inflammatory M2 type rather than the pro-inflammatory M1 type [21]. Adropin decreased fat accumulation in adipose tissues and reduced adipose tissue macrophage infiltration, thus decreasing inflammation [22,23].

In addition, its antioxidant effect is determined through its ability to mitigate hepatocyte injury and increase glutathione levels in mice with nonalcoholic steatohepatitis [20]. Moreover, it increased superoxide dismutase (SOD) and reduced malondialdehyde (MDA) levels in rat cardiomyoblast cells exposed to ischemia/reperfusion injury [19]. Additionally, Choi and Yim [24] detected a non-significant correlation between adropin and MDA in type 2 diabetic patients.

**Figure 1:**
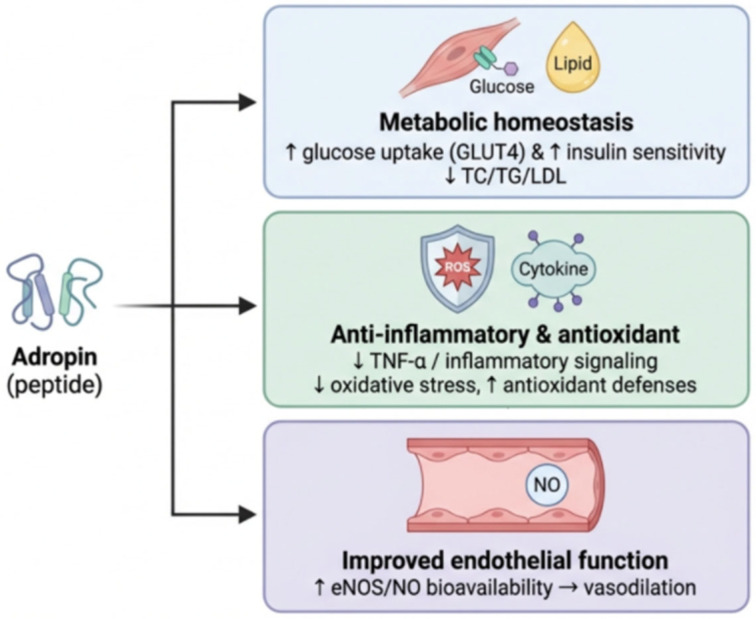
Adropin-Mediated Improvements in Metabolism, Oxidative Stress, and Vascular Function. Adropin promotes metabolic homeostasis by increasing GLUT4-mediated glucose uptake and insulin sensitivity, accompanied by reductions in total cholesterol (TC), triglycerides (TG), and low-density lipoprotein cholesterol (LDL-C). It attenuates inflammation and oxidative stress by decreasing tumor necrosis factor-α (TNF-α), reactive oxygen species (ROS), and increased antioxidant defenses. In addition, adropin improves endothelial function by enhancing endothelial nitric oxide synthase (eNOS) activity and nitric oxide (NO) bioavailability, leading to vasodilation. Created with BioRender.com. Retrieved from https://BioRender.com/6t529z1 (accessed on 1 March 2026).

Adropin also regulates endothelial function; diminished adropin levels correlate with endothelial dysfunction, as evidenced by the flow-mediated dilation (FMD) tests in metabolic syndrome patients [25]. Adropin up-regulates nitric oxide synthase in the endothelium and increases nitric oxide bioavailability, which enhances dilation of blood vessels [26] (Figure 1).

## 3. Adropin Effects on Different Organs

Adropin plays an important role in maintaining normal physiological homeostasis across multiple organs. Adropin concentration in the brain is highest and is necessary for the proper development and function of the mouse’s cerebellum. Adropin knockout mice showed decreased physical activity, abnormal coordination of movements, and impaired synaptic formation in the cerebellum [2].

In addition, adropin has a protective role in the brain. Adropin administration to rats’ brains exposed to hypoxia and low glucose conditions in vitro caused a decrease in brain endothelial cell permeability [27]. Nasal adropin administration to mice after intracerebral hemorrhage reduced brain water content, improved neurological function, and preserved the blood–brain barrier [28].

Moreover, Adropin succeeded in restoring the main cardiac metabolic function in cardiometabolic heart failure with preserved ejection fraction, as presented by reducing fibrosis, diastolic dysfunction, and cardiomyocyte hypertrophy [29].

Regarding the genital organs, recent research highlights the critical regulatory role of adropin in the female reproductive system. In the ovaries, the corpus luteum, granulosa cells, and theca-interstitial cells express adropin [30]. It acts as a protective agent by enhancing antioxidant defense, promoting follicular cell survival, and simultaneously reducing oxidative stress-induced cellular damage [30]. Furthermore, this hormone works together with the human chorionic gonadotropin (hCG) hormone to further stimulate ovarian steroidogenesis and survival through facilitating the expression of steroidogenic acute regulatory protein (StAR), 3β-hydroxysterol dehydrogenase (3β-HSD), and aromatase proteins and diminishing the BAX/BCL2 ratio [30].

Experimentally, restoring normal adropin levels helped normalize irregular menstrual cycles and reduce excessive testosterone production in polycystic ovary syndrome (PCOS) mice by suppressing overactive ovarian enzymes [31].

In addition, adropin’s relationship to testicular function has recently emerged; so far, few studies have reported an association between adropin and testosterone secretion and spermatogenesis. This review focuses on illuminating the role of adropin in regulating testicular function and describing its mechanism of action and highlights possible future interventions that could further enhance the benefits of adropin in improving testicular function.

## 4. Methods

We searched published studies in PubMed, Scopus, and ScienceDirect databases using a combination of relevant keywords, including “adropin”, “testis”, “spermatogenesis”, “testosterone”, “luteinising hormone (LH)”, “follicle-stimulating hormone (FSH)”, and “gonadotropin-releasing hormone (GnRH)”. All studies relevant to the topic and published up to 16 March 2026 were included.

## 5. Effect of Adropin on the Hypothalamic–Pituitary–Testicular Axis

Based on our screening, only one study has evaluated the effect of adropin on the hypothalamic–pituitary–testicular axis in male rats through injecting adropin intraperitoneally in two different doses, a small dose, 4 μg/kg/day for 10 days, and a larger dose, 40 μg/kg/day for 10 days, and measured levels of GnRH, FSH, LH, testosterone, inhibin B, and activin A, in addition to histological and immunohistochemical analysis of cerebral cortex and testicular tissues [32].

Researchers observed reduced circulating LH levels in the high-dose adropin group, whereas they found no statistically significant difference between the low-dose adropin group and the control group. Regarding circulating FSH levels, adropin administration did not produce a statistically significant difference between groups [32]. In rats with PCOS, adropin administration restored normal LH values and increased FSH levels [33]. In contrast, researchers observed no significant correlations between adropin and LH or FSH in PCOS women [34,35]. However, in the study by Yildirim et al. [36], they detected a significant negative correlation between adropin and FSH in PCOS women, whereas LH showed a non-significant negative correlation with adropin.

Concerning adropin relation to testosterone, administration of adropin to pre-pubertal and adult mice enhanced testosterone secretion by increasing the expression of steroidogenic enzymes [37,38]. In addition, intraperitoneal adropin administration in Wistar albino rats increased circulating testosterone level in a dose-dependent manner [32].

Interestingly, hypothalamic GnRH detected by immunohistochemistry exhibited increased immunoreactivity in the group treated with the low-dose adropin and a further increase in the high-dose adropin group [32].

Kisspeptin is a neuropeptide that stimulates the release of GnRH from the hypothalamus [39]. Hypothalamic kisspeptin exhibited higher values in adropin-treated rats than controls, which were higher in the high-dose adropin group than the low-dose adropin-treated group [32].

Researchers observed increased hypothalamic kisspeptin and GnRH immunoreactivity in adropin-treated rats; however, circulating LH levels were reduced in the high-dose group. This apparent discrepancy may be explained by the fact that immunoreactivity reflects tissue peptide content rather than the dynamics of neuroendocrine secretion, including pulse frequency and amplitude, which are critical determinants of LH release. In addition, they measured LH concentrations at a single time point after 10 days of treatment, which does not capture the pulsatile and phase-dependent nature of hypothalamic–pituitary signaling [32].

Activins and inhibins are two peptide hormones of the transforming growth factor-β (TGF-β) superfamily that play critical roles in regulating FSH secretion, cellular proliferation, and embryonic development [40].

Rats treated with adropin exhibited lower values of activin A in a dose-dependent manner. At the same time, inhibin B showed higher circulating levels in the low-dose adropin-administered rats than in controls, and levels were much higher in the high-dose adropin group [32].

Findings of low activin A and high inhibin B in relation to high kisspeptin expression are controversial, as activin A and inhibin A have been reported to influence Kiss1 gene expression. In contrast, inhibin B suppresses this expression [41], which is opposite to the findings of Eraslan et al. [32]. In addition, they detected no statistically significant differences in FSH levels in adropin-treated groups, although activin A was low and inhibin B was high [32]. However, it is known that activin A stimulates pituitary synthesis and secretion of FSH, whereas inhibin B antagonizes activin’s action and selectively suppresses FSH release [40,42]. These controversial results could reflect temporary dissociation between circulating hormones and central gene regulation or could be partially affected by the negative feedback effect of testosterone on various compartments of the hypothalamic–pituitary–testicular axis [32].

These overall results suggest both a central and a peripheral role for adropin in stimulating testosterone secretion, which warrants further evaluation.

Cerebral cortical histological sections showed normal neuronal morphology with no areas of hemorrhage, necrosis, or inflammation in both rat groups treated with adropin, which was totally similar to the control group [32].

Histological evaluation of testicular tissue from adropin-treated pre-pubertal and adult mice showed no major changes compared to control mice [37,38]. Moreover, histological sections of testicular tissue from adropin-treated rats (4 and 40 μg/kg/day administered intraperitoneally for 10 days) showed normal testicular architecture, with normal tubular organization and preserved germinal epithelium thickness. Additionally, both adropin-treated groups exhibited increased testicular expression of superoxide dismutase 1, an antioxidant enzyme, in both adropin-treated groups, with higher expression reported in the high-dose adropin group [32]. Increased superoxide dismutase 1 expression was observed primarily in spermatocytes and Leydig cells, suggesting a possible functional role for adropin in enhancing spermatogenesis and testosterone production. However, parameters indicative of testicular reproductive function were not detected in the study by Eraslan et al. [32].

Although cerebral and testicular histological findings showed normal architecture, these results should not be used as an indicator of adropin safety, as observing cells with H&E only lacks evaluation of cellular oxidative stress, possible apoptosis, or ultrastructural injury; thus, more comprehensive research is required to ensure adropin’s safety.

Interestingly, adropin facilitated weight loss, whereas rats in the control group showed modest weight gain. In contrast, adropin-treated groups showed decreased body weight, with the highest decrease observed in the high-dose adropin group [32]. This could be related to adropin’s ability to enhance insulin sensitivity and decrease plasma lipids, oxidative stress, and feeding behavior [1,43,44].

## 6. Adropin Gene, Receptor, and Protein Expression in the Testis

Adult mouse testis expresses the Enho gene [45]. Testicular expression of the Enho gene suggests that adropin is locally synthesised in the testis. Maximum GPR19 expression was observed in the pre-pubertal testis, followed by a sharp decline in puberty, a significant increase in the reproductive stage, and a further decline with aging [37].

The orphan GPR19 is highly expressed in the adult mouse testis [6], which is suggested to be the possible site of adropin action [45,46,47]. This directed attention towards discovering new functions of adropin through its action on the testis.

GPR19 expression varied across testicular cell types at different developmental stages, with intense expression observed in Leydig cells and gonocytes in the infantile mouse testis. During the pre-pubertal, pubertal, and reproductive stages, GPR19 was strongly expressed in mouse pachytene spermatocytes and showed mild to moderate expression in Leydig cells, primary, and secondary spermatocytes. This expression showed an age-dependent decline in aged mice [37]. GPR19 expression in Leydig cells and nearby germ cells suggests an autocrine as well as a paracrine action of adropin [45].

**Figure 2:**
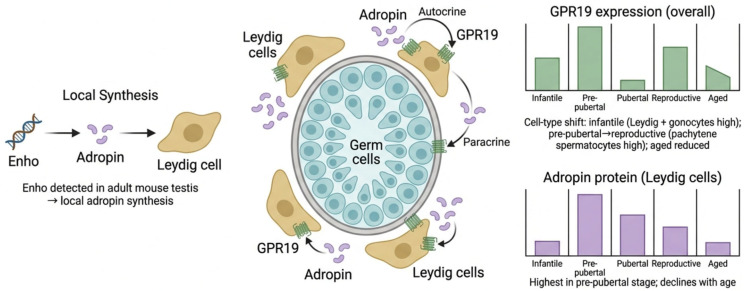
Local adropin synthesis and GPR19 expression during mouse testis development. Enho expression in the adult mouse testis suggests local synthesis of adropin, primarily in Leydig cells. Locally produced adropin acts through its putative receptor GPR19 via autocrine signaling in Leydig cells and paracrine signaling toward adjacent germ cells within the seminiferous tubules. Developmental analysis indicates that overall GPR19 expression in mouse testes varies across life stages, with dynamic cell-type distribution. During infancy, GPR19 appears to be highly expressed in Leydig cells and gonocytes followed by a shift toward pachytene spermatocytes during the subsequent stages. Higher expression appears to be present during the pre-pubertal stage, followed by a sharp decline in puberty, a significant increase in the reproductive stage, and a reduction in aged testes. Adropin protein levels in mice’s Leydig cells peak during the pre-pubertal stage and progressively decline with aging. These findings suggest a possible stage-specific regulation of the adropin–GPR19 axis in testicular development and function. However, the results shown represent inferred mechanisms based on limited preclinical animal studies and have not yet been fully validated experimentally. Created with BioRender.com. Retrieved from https://BioRender.com/73jc24w (accessed on 1 March 2026).

Researchers detected adropin protein expression in Leydig cells at all developmental stages. They observed the lowest expression levels in the infantile mouse testis and the highest levels in the prepubertal stage, suggesting a potential role of adropin in pubertal development. This is followed by a progressive decline of adropin levels with advancing age, reflecting age-associated reduction in testicular function [45] (Figure 2).

## 7. Adropin Enhances Testicular Testosterone Synthesis

Adropin binds to receptors expressed in Leydig cells of the testis, modulating testosterone secretion. Leydig cells of the testis are the primary source of testosterone [48]. Testosterone, the male sex hormone, is essential for the development of male organs and sex characteristics, as well as for healthy sperm production. It preserves the integrity of the blood-testis barrier and stabilizes the physical connections between Sertoli cells and developing germ cells. It is also necessary for meiotic progression and spermatid maturation [49,50,51].

Transport of cholesterol into mitochondria by StAR proteins is the first step in the production of testosterone. Cytochrome P450-SCC in the mitochondria converts cholesterol into pregnenolone, which is subsequently transported to the smooth endoplasmic reticulum, where it is converted into testosterone by vital steroidogenic enzymes such as 3β-HSD, cytochrome P450 17A1, and 17β-hydroxysteroid dehydrogenase (17β-HSD) [52,53].

In obese men, researchers detected a positive association between serum adropin and serum total testosterone levels; in addition, they reported that adropin might be considered a predictive risk factor for testosterone, which suggested a possible role of adropin in modifying testosterone production [54].

In men with erectile dysfunction induced by non-alcoholic fatty liver disease (NAFLD), a study detected statistically significant positive correlations between the Arabic version of the International Index of Erectile Function (ArIIEF-5) score, adropin, and testosterone. Furthermore, adropin was reported to be a strong independent predictor of the ArIIEF-5 score. This highlights the pivotal role of adropin in erectile dysfunction associated with NAFLD [55].

### 7.1. In Vitro Effects of Adropin on Testicular Testosterone Synthesis in the Presence and Absence of Insulin

To identify the direct adropin action on the testis, testicular slices were cultivated with two different doses of adropin (10 and 100 ng/mL). Interestingly, adropin inhibited testosterone synthesis, as manifested by decreased testicular testosterone levels at the higher dose. This decrease in testosterone levels occurred by down-regulating the expression of P450-SCC, 3β-HSD, and 17β-HSD. However, adropin failed to significantly decrease StAR expression. Regarding P450-SCC and 17β-HSD testicular expressions, the 100 ng/mL adropin reduced their expression levels to a lower value than the 10 ng/mL adropin. In contrast, 3β-HSD showed minimal differences in expression levels at both adropin doses [45]. In agreement with this, Stelcer et al. [56] observed inhibited adrenocortical steroidogenesis in a human adrenocortical carcinoma cell line following incubation with adropin. They attributed this inhibition to adropin binding to GPR19, which activates the TGF-β-dependent pathway and subsequently downregulates StAR expression.

Adding insulin (5 μg/mL) to cultured testicular tissues treated with adropin (10 and 100 ng/mL) reversed the effects obtained with adropin alone. Specifically, GPR19 expression increased, along with testosterone synthesis, through up-regulating StAR and key steroidogenic enzymes, including P450-SCC, 3β-HSD, and 17β-HSD. In addition, insulin receptor expression was elevated [45].

Consistent with these findings, He et al. [57] have shown that treating diabetic rats with adropin significantly increased serum insulin levels. Accordingly, it has been proposed that adropin enhances testosterone production by increasing insulin sensitivity and promoting insulin-mediated testicular testosterone synthesis [45].

Furthermore, exposure of mouse Leydig cells to insulin prior to LH stimulation increased testosterone synthesis compared with cells not pre-treated with insulin [58]. Additionally, deletion of the insulin receptor and the insulin-like growth factor 1 receptor in steroidogenic cells in mice markedly reduced testicular size and impaired testicular testosterone synthesis [59].

**Figure 3:**
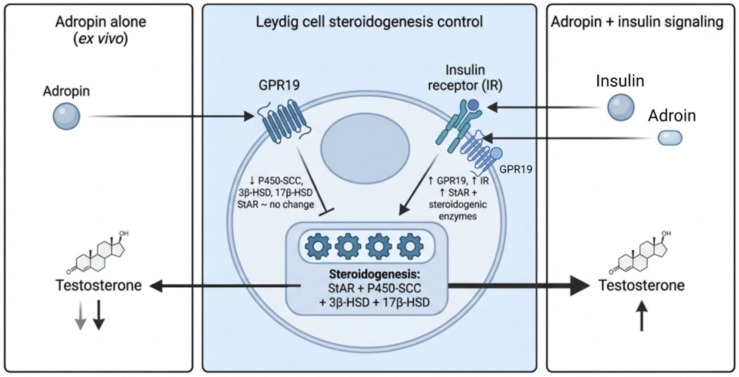
Context-dependent effects of adropin on Leydig cell testosterone synthesis. Schematic illustrating the differential effects of adropin on Leydig cell steroidogenesis depending on metabolic context. Under ex vivo conditions (culturing adult mice testicular tissue with adropin alone), adropin binds to GPR19 and is associated with reduced expression of key steroidogenic enzymes, including 17β-hydroxysteroid dehydrogenase (17β-HSD), P450 side-chain cleavage enzyme (P450scc), and 3β-hydroxysteroid dehydrogenase (3β-HSD) with no significant change in steroidogenic acute regulatory protein (StAR), resulting in decreased testosterone production. In contrast, in the presence of insulin signaling, adropin acts in conjunction with insulin receptor (IR) activation, leading to increased GPR19 and IR expression, upregulation of StAR and downstream steroidogenic enzymes, and enhanced testosterone synthesis. This model illustrates a possible context-dependent, insulin-sensitive modulation of Leydig cell function by adropin. However, this association represents an inferred mechanism based on limited animal evidence. Created with BioRender.com. Retrieved from https://BioRender.com/cfblviv (accessed on 1 March 2026).

Taken together, these findings suggest that insulin, through binding to receptors expressed in Leydig and Sertoli cells, enhances testosterone production [60] by up-regulating steroidogenic enzyme expression [45] (Figure 3).

However, although existing data suggest insulin-dependent modulation of steroidogenesis, the precise molecular pathway has not been fully elucidated.

Interestingly, culturing testicular tissue with the lower dose of adropin (10 ng/mL) and 5 μg/mL insulin resulted in higher GPR19 and insulin receptor expression, as well as higher testicular testosterone levels, compared to tissues cultured with 100 ng/mL adropin and 5 μg/mL insulin. Additionally, StAR, 3β-HSD, and 17β-HSD expression levels were higher in testicular tissues cultured with the lower adropin concentration when compared to tissues cultured with the higher adropin concentration. In contrast, P450-SCC testicular expression revealed the opposite results [45].

### 7.2. Effect of Intratesticular Adropin Administration in Adult Mice on Testosterone Synthesis

Adropin was directly injected into the testes of adult mice (0.5 μg/testis) to assess its direct in vivo effects on testicular function. GPR19 expression and steroidogenic enzyme expression, including StAR, P450-SCC, 3β-HSD, and 17β-HSD, were up-regulated, increasing testicular testosterone secretion [38].

Although adropin inhibited testosterone synthesis in ex vivo testicular slice preparations, its intratesticular administration in vivo was associated with an overall stimulatory effect on testosterone production. This apparent discrepancy likely reflects fundamental differences between the two experimental models. In the ex vivo setting, testicular tissue is isolated from systemic endocrine, metabolic, and paracrine influences, thereby revealing the direct local action of adropin on Leydig cell steroidogenic activity. In contrast, the in vivo testicular environment preserves physiological interactions, such as insulin signaling, which may modulate or counterbalance the direct inhibitory effects observed in isolated tissue.

Further research is needed to elucidate the precise mechanisms of adropin action on the testis in vivo, as well as to investigate its interactions with other physiological systems that may influence or modify its functional effects.

### 7.3. Effect of Intratesticular Adropin Administration in Pre-Pubertal Mice on Testosterone Synthesis

Moreover, adropin administered as intra-testicular injections (0.5 and 1.5 µg/testis) in pre-pubertal testes significantly increased testicular testosterone levels compared to the vehicle group at both doses, with only a minimal difference observed between them [37].

In parallel, androgen receptor expression increased with increasing adropin dose. Steroidogenic enzymes exhibited dose-dependent alterations: StAR was significantly increased at the higher dose but did not reach statistical significance at the lower dose. In contrast, 3β-HSD and 17β-HSD expression significantly elevated at both doses, with 3β-HSD showing a greater increase at the lower dose and 17β-HSD at the higher dose. While the P450-SCC protein showed a non-significant elevation at either dose. The increased testosterone production during the pre-pubertal period may reflect the elevated expression of adropin and GPR19 observed during this stage compared to other developmental periods. Collectively, these results indicate that adropin may contribute to pubertal testicular maturation, a process that warrants further investigation [37].

### 7.4. Effect of Intraperitoneal Adropin Administration in Adult Rats on Testosterone Level

Testosterone levels determined after high-dose adropin administration intraperitoneally (40 μg/kg/day for 10 days) into male Wistar albino rats were significantly increased compared to controls, while the low-dose adropin group (4 μg/kg/day for 10 days) showed an intermediate testicular value between the control and the high-dose adropin groups, suggesting a dose-dependent increase in circulating testosterone levels with adropin administration [32].

### 7.5. Effect of Adropin Administration in Diabetic Mice on Testosterone Synthesis

According to some animal studies, adropin shares in the regulation of glycolipid metabolism and the enhancement of insulin sensitivity [1,4,13,14]. Furthermore, in type 2 diabetic patients, serum adropin levels were reduced and correlated with fasting blood glucose levels [61]. However, the association between adropin and testicular testosterone in diabetes remains unclear. One study investigated this by administering adropin intraperitoneally (450 nmol/kg body weight) to diabetic mice to assess its potential effects on testicular function. Adropin reduced blood glucose levels and insulin resistance and increased serum insulin levels as well as testicular insulin receptor expression. In addition, adropin elevated testicular testosterone levels that were diminished by diabetes by upregulating the expression of steroidogenic enzymes, StAR, P450-SCC, 3β-HSD, and 17β-HSD [62].

## 8. Adropin Stimulates Spermatogenesis

Spermatogenesis, the process of sperm formation, is a highly coordinated process that involves testicular cells, sex steroids, gonadotropins, and other hormones and proteins working together to produce fully developed spermatozoa [63].

Spermatogenesis is divided into three steps: mitosis, meiosis, and spermiogenesis. Mitotic division of spermatogonial stem cells (2N:2C) gives rise to differentiating spermatogonia. After DNA replication during the S phase, primary spermatocytes (2N:4C) are formed, which subsequently undergo meiosis I to form secondary spermatocytes (1N:2C). These cells then enter meiosis II, producing spermatids (1N:1C), which further undergo spermiogenesis, during which spermatids are specialized into mature spermatozoa [64].

### 8.1. Spermatogenesis in Testicular Tissues Cultured with Adropin

Adropin cultured with testicular tissues (10 and 100 ng/mL) enhanced testicular germ cell proliferation and survival in a dose-dependent manner through increasing proliferating cell nuclear antigen (PCNA) expression. This nuclear protein is essential for DNA replication and repair. As well as increasing phosphorylated extracellular signal-regulated kinases 1 and 2 (pERK1/2), which are indicative of ERK signalling activation involved in survival and proliferation [45].

Culturing testicular tissues with adropin (10 and 100 ng/mL) and insulin (5 μg/mL) exhibited increased PCNA and pERK1/2 expressions, but with higher levels detected in testicular tissues cultured with the 10 ng/mL adropin compared to tissues cultured with the higher adropin concentration [45]. Insulin could stimulate spermatogenesis in mouse testes via binding to its testicular receptors [65].

### 8.2. Effect of Intratesticular Injection of Adropin on Spermatogenesis in Adult Mice

Testicular histological sections of adult mice that received intratesticular adropin (0.5 μg/testis) exhibited no major histological alteration compared to normal mice testes with well-organized seminiferous tubules but increased PCNA expression and sperm count [38].

### 8.3. Effect of Adropin on Spermatogenesis in the Pre-Pubertal Testis

Histological sections after adropin administration directly in pre-pubertal testes at two doses, 0.5 μg/testis and 1.5 μg/testis, showed well-organised seminiferous tubules associated with various stages of developing germ cells and a dose-dependent increase in the expression of PCNA, a cell proliferation marker. Immunohistochemistry revealed high PCNA expression in spermatogonial cells at both adropin doses and lower PCNA expression in spermatocytes, while the normal pre-pubertal testis revealed PCNA expression in spermatogonia only, but not in spermatocytes. The observed increased expression of PCNA in spermatogonia and spermatocytes of adropin-treated testis suggests the role of adropin in the proliferation and differentiation of spermatogonia into successive germ cells [37].

### 8.4. Adropin Enhances Spermatogenesis in Hyperglycemic Mice

In hyperglycemic mice, intraperitoneal adropin administration increased the number of spermatids and sperm count. It restored the altered testicular histological structure induced by diabetes, revealing well-formed seminiferous tubules containing normal cells, including spermatogonia, spermatocytes, spermatids, Sertoli cells, and Leydig cells [62].

**Figure 4:**
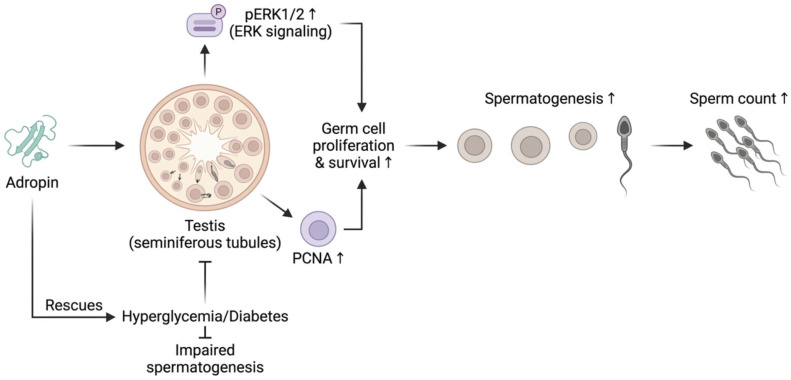
Adropin promotes germ cell proliferation and spermatogenesis via ERK signaling. Culturing testicular tissues with adropin activated ERK signaling, as indicated by increased phosphorylation of ERK1/2 (pERK1/2). This activation is associated with upregulation of proliferating cell nuclear antigen (PCNA), reflecting enhanced germ cell proliferation and survival. Consequently, spermatogenesis is thought to be increased, leading to a higher sperm count. Additionally, adropin ameliorates hyperglycemia/diabetes-associated impairment of spermatogenesis in hyperglycemic mice, suggesting a possible protective role in metabolically compromised conditions. However, the mentioned pathway is based primarily on limited preclinical evidence and has not yet been fully validated experimentally. Created with BioRender.com. Retrieved from https://BioRender.com/px0z2lg (accessed on 1 March 2026).

Adropin-treated hyperglycemic mice exhibited higher PCNA spermatogonial expression compared to non-treated hyperglycemic mice. This suggests the role of adropin in restoring germ cell proliferation that was diminished by diabetes [62] (Figure 4).

## 9. Adropin Effect on Different Testicular Germ Cell Kinetics

Using fluorescence-activated cell sorting analysis, accelerated germ cell differentiation was detected in adult mice, with increased numbers of spermatids (1C) and primary spermatocytes (4C), while decreased numbers of spermatogonia (2C) were observed [38]. Reduced spermatogonia indicates a higher rate of germ cell proliferation [66]. Additionally, adropin administration increased the 1C/2C and 4C/2C ratios, suggesting adropin’s ability to stimulate the overall turnover of testicular cells [38].

Pre-pubertal testes treated with a high dose of adropin (1.5 μg/testis) exhibited an increased number of spermatids (1C) and a 1C/2C ratio. In contrast, a low dose of adropin (0.5 μg/testis) failed to increase the number of spermatids (1C) or the 1C/2C ratio to statistical significance. Additionally, neither the high nor the low adropin dose significantly changed the number of spermatogonia (2C) and primary spermatocytes (4C) compared with normal. Whereas 1C to 4C cells showed a slight increase that did not reach statistical significance, no change in the 4C to 2C ratio was observed. Increased spermatid number with the higher adropin dose could suggest a possible role of adropin in germ cell proliferation and sperm formation [37].

Furthermore, adropin elevated the reduced number of spermatids (1C), spermatogonia (2C), and primary spermatocytes (4C) in diabetic mice, suggesting the possible beneficial effect of adropin treatment in restoring germ cell proliferation in diabetic mice [62].

## 10. Adropin Alleviates Apoptosis in the Testis

Adropin cultured with testicular tissue reduced testicular germ cell apoptosis by increasing the anti-apoptotic marker B-cell lymphoma-2 (Bcl2). Moreover, testicular tissues cultivated with adropin and insulin showed higher Bcl2 expression levels, with higher expression detected in tissues cultured with 100 ng/mL adropin than in tissues cultured with 10 ng/mL adropin [45].

**Figure 5:**
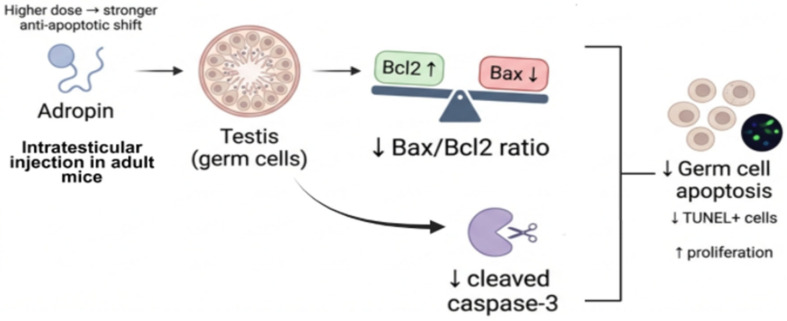
Adropin reduces germ cell apoptosis through modulation of the Bax/Bcl2 pathway. Adult mouse testicular tissue cultured with adropin and insulin exhibited a dose-dependent anti-apoptotic shift in germ cells through increasing the expression of the anti-apoptotic protein Bcl2. Intratesticular injection of adropin in adult mice increased the anti-apoptotic protein Bcl2 expression and decreased the pro-apoptotic protein Bax, resulting in a reduced Bax/Bcl2 ratio. This shift is associated with decreased cleavage of caspase-3 and reduced germ cell apoptosis, as evidenced by fewer TUNEL-positive cells. Additional research is needed to support these findings, as they are based on limited preclinical studies. Created with BioRender.com. Retrieved from https://BioRender.com/jxh3i1c (accessed on 1 March 2026).

In addition, intra-testicular adropin administration in adult mice inhibited apoptosis and enhanced cell proliferation by reducing the expression of pro-apoptotic proteins (Bax, caspase 3, and cleaved caspase 3), decreasing TUNEL-positive cells, increasing Bcl2 protein expression, and decreasing the Bax/Bcl2 ratio [38] (Figure 5).

Moreover, intratesticular adropin injection into pre-pubertal mice testes at two doses (0.5 and 1.5 μg/testis) significantly reduced the Bax/Bcl2 ratio, although both Bcl2 and Bax protein expressions were elevated. Whereas caspase-3 protein expression and TUNEL-positive cells declined, which supports diminished apoptosis in pre-pubertal testes treated with adropin [37]. Researchers found that cells with a low Bax/Bcl2 ratio were less vulnerable to apoptotic injury than those with a high Bax/Bcl2 ratio [67]. Interestingly, the expression levels of Bax, Bcl2, and caspase-3 decreased with increasing adropin doses [37].

Furthermore, adropin alleviated increased apoptosis in diabetic mice by inhibiting Bax and cleaved caspase-3 expression, decreasing the testicular Bax/Bcl2 ratio, and decreasing TUNEL-positive cells [62].

## 11. Adropin Mitigates Oxidative and Nitrosative Stresses

Intratesticular adropin administration to adult mice elevated the testicular expression of antioxidant enzymes such as heme oxygenase-1 (HO-1), SOD, and catalase while reducing MDA and nitric oxide levels [38].

Additionally, it increased the expression and intranuclear translocation of nuclear factor erythroid 2–related factor 2 (NRF2), a key transcription factor that regulates cellular antioxidant defense, whereas it decreased the expression and intranuclear translocation of the nuclear factor kappa-light-chain-enhancer of activated B cells (NF-κB), a transcription factor that amplifies cellular inflammation and stress responses [38].

This adropin-mediated activation of the NRF2/HO-1 signaling axis is not exclusive to testicular tissue; it may also serve as a protective mechanism against oxidative damage in mouse models of nonalcoholic steatohepatitis and heart failure [20,68].

## 12. Conclusions

In conclusion, adropin, a protein that is synthesised in the testis and signals through its receptor GPR19, is suggested to be involved in the regulation of testicular function. Adropin may enhance testosterone secretion, potentially by increasing steroidogenic enzyme activity. In addition, it might be associated with germ cell proliferation and sperm formation. It is also suggested to exert testicular anti-apoptotic and antioxidant effects, which could help protect testicular cells from damage and support overall reproductive health.

Adropin levels appear to be higher prior to puberty, which may contribute to normal testicular development, testosterone production, and spermatogenesis.

In contrast, reduced levels with aging may be associated with declining testicular function. However, the evidence supporting its function is limited and largely restricted to a narrow range of preclinical animal models and relatively short-term interventions. Further research is required to support this evidence and to elucidate the possible underlying mechanisms clearly.

## 13. Future Work

A lot remains to be discovered; future research is required to fully understand the exact underlying molecular mechanism of adropin action in the testis, hypothalamus, and pituitary and to further identify the possible anti-inflammatory action of adropin in both normal and diseased testes. Experimental studies in normal and hyperglycemic animal models have reported improvements in testicular function following adropin administration. However, these findings remain preclinical, and further studies are required to clarify its mechanisms of action, evaluate its safety profile, and determine whether it has any relevance in reproductive endocrine regulation. Its potential involvement in conditions associated with altered testosterone levels, impaired spermatogenesis, or delayed puberty remains to be investigated.

## Data Availability

No new data were created or analyzed in this study. Data sharing is not applicable to this article.
